# Grafting with rootstocks induces extensive transcriptional re-programming in the shoot apical meristem of grapevine

**DOI:** 10.1186/1471-2229-13-147

**Published:** 2013-10-02

**Authors:** Sarah Jane Cookson, Nathalie Ollat

**Affiliations:** 1 INRA, ISVV, EGFV, UMR 1287, Villenave d’Ornon, F-33140, France

## Abstract

**Background:**

Grafting is widely used in the agriculture of fruit-bearing crops; rootstocks are known to confer differences in scion biomass in addition to improving other traits of agricultural interest. However, little is known about the effect of rootstocks on scion gene expression. The objective of this study was to determine whether hetero-grafting the grapevine variety *Vitis vinifera* cv. 'Cabernet Sauvignon N’ with two different rootstocks alters gene expression in the shoot apex in comparison to the auto-grafted control. Cabernet Sauvignon was hetero-grafted with two commercial rootstock genotypes and auto-grafted with itself. Vigor was quantified by measurements of root, stem, leaf and trunk biomass. Gene expression profiling was done using a whole genome grapevine microarray; four pools of five shoot apex samples were harvested 4 months after grafting for each scion/rootstock combination.

**Results:**

The rootstocks increased stem biomass or conferred increased vigor by the end of the first growth cycle. Globally hetero-grafting two different genotypes together triggered an increase in shoot apex gene expression; however no genes were differentially expressed between the two hetero-grafts. The functional categories related to DNA, chromatin structure, histones, flavonoids and leucine rich repeat containing receptor kinases were the most enriched in the up-regulated genes in the shoot apex of hetero-grafted plants.

**Conclusions:**

The choice of rootstock genotype had little effect on the gene expression in the shoot apex; this could suggest that auto- and hetero-grafting was the major factor regulating gene expression.

## Background

Grafting is widely used in the agriculture of fruit crops. Rootstocks are selected to provide resistance to soil-borne pests and diseases, to increase tolerance to environmental stresses and to improve crop productivity and/or quality (e.g. as reviewed by [1,2]). In viticulture, grafting is primarily used to facilitate grapevine growth in soils infected with the phylloxera, a soil-dwelling insect pest introduced to Europe from America (e.g. [3]). The use of rootstocks, in which the entire root system of a plant is replaced, has a profound effect on scion development. Rootstocks are known to alter various physiological processes in the scion such as vigor or biomass accumulation [4], fruit quality [5] and response to abiotic stresses (e.g. to water deficit [6] and salinity [7]).

Despite the widespread use of grafting, we know little of how grafting with rootstocks confers differences to the vigor of the scion. Rootstock conferred vigor in horticulture is generally described in terms of shoot biomass accumulation, i.e. stem biomass, yield or biomass allocation within the plant (such as the ratio between yield and stem biomass). Various hypotheses explaining rootstock conferred vigor have been proposed including alterations in nutrient and water movement, hormone concentrations and the anatomy of the graft union (as reviewed by [2,8]). In apple orchards, the rootstocks used have dramatic dwarfing effects on the scion, reducing trunk diameter by up to 70% e.g. [9]. In cherry trees, rootstock-induced dwarfing (reducing stem length by 25%) is caused by differential cessation of terminal meristem growth e.g. [10]. Commercial rootstocks used in viticulture have much smaller effects on scion vigor. They are not associated with dwarfing phenotypes observed in fruit trees, but grapevine rootstocks are still important for fruit quality and yield. Although vigor control mechanisms of different rootstocks are not necessarily the same for different scions, there is some degree of consistency with most commercial rootstock genotypes, being defined by viticulturists as high, moderate or low vigor e.g. [3].

Gene expression studies have been used to shed light on the mechanisms behind rootstock conferred vigor in fruit trees. Gene expression in the shoot tip of apple trees was studied in response to a range of rootstock genotypes to correlate shoot tip expression to plant stature [11]. The Gene Ontology (GO) terms enriched in the differentially expressed genes were response to stimulus and response to abiotic and biotic stress as well as unknown biological processes and other biological processes. Jensen et al. [11] identified 116 genes whose expression levels were correlated with plant size; the genes most strongly correlated with trunk cross sectional diameter were a sorbitol dehydrogenase, a homeobox-leucine zipper protein and a hevein-like protein. Similarly, Prassinos et al. [10] used cDNA amplified fragment length polymorphism to compare the effect of a dwarfing and a semi-vigorous rootstocks on gene expression in the scion and identified the differential regulation of a number of transcription factors and genes involved in signaling processes. However, auto-grafted control plants were absent from the studies of Jenson et al. [11] and Prassinos et al. [10], therefore the effect of grafting with a non-self rootstock on the gene expression of the scion remains to be determined.

Our objective was to study the effects of grafting on gene expression in the shoot apical meristem of grapevine *Vitis vinifera* cv. 'Cabernet Sauvignon N’ (CS) heterografts (with two different commercial rootstock genotypes originating from American *Vitis spp.*) and the auto-grafted control (CS grafted with CS). The rootstock genotypes chosen were 'Riparia Gloire de Montpellier’ (RG) and '1103 Paulsen’ (1103P), these genotypes are known to confer low and high scion vigor respectively. Our sampling strategy was designed to minimize the secondary effects related to rootstock physiology, such as, differences in plant water status, gas exchange and transpiration. Therefore, shoot apical meristems were harvested at the end of the night when presumably plants are in a stable environment to reduce confusion of the direct and indirect effects of grafting with rootstocks. Genes from the functional categories DNA, chromatin structure, histones, flavonoids and leucine rich repeats (LRR) were enriched in the up-regulated genes.

## Results

In horticulture, rootstocks are often selected for their ability to alter the growth of the scion, or to confer differences in scion vigor. In viticulture, vigor is typically quantified by stem pruning weight at the end of the growing cycle and, in this work, conferred vigor was also quantified by using measurements of dry stem biomass. The objective was to associate differences in rootstock conferred vigor (i.e. stem biomass) with differences in gene expression in the shoot apex. Shoot apex samples were harvested four months after grafting when presumably the stress associated with grafting and the formation of a successful graft union has been overcome. Some of the genes differentially expressed in the microarray data were confirmed by qPCR (Additional file 1).

### Rootstock conferred vigor effects on biomass accumulation in grafted grapevine

**Figure 1:**
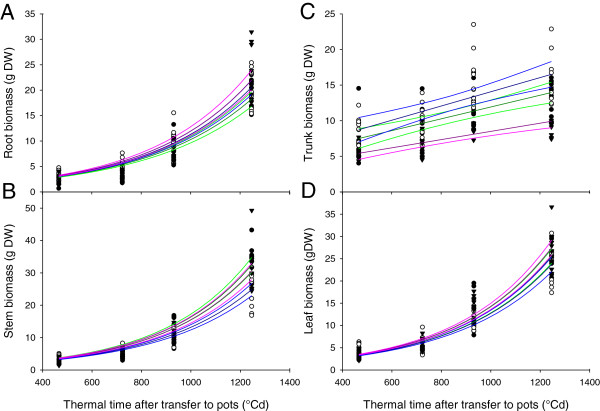
**Rootstock effects on biomass accumulation in grapevine during the first growth cycle.** Biomass accumulation was fitted to curves and 95% confidence intervals were calculated, for panels **A**, **C** and **D** the curve fitted was y = e^ax^ and for B it was y = mx + C. Biomass in the root **(A)**, trunk **(B)**, stem **(C)** and leaves **(D)** of Cabernet Sauvignon grafted with the rootstocks 1103 Paulsen (filled circles, green lines) and Riparia Gloire de Montpellier (filled triangles, pink lines) and the auto-grafted control (open circles, blue lines).

**Figure 2:**
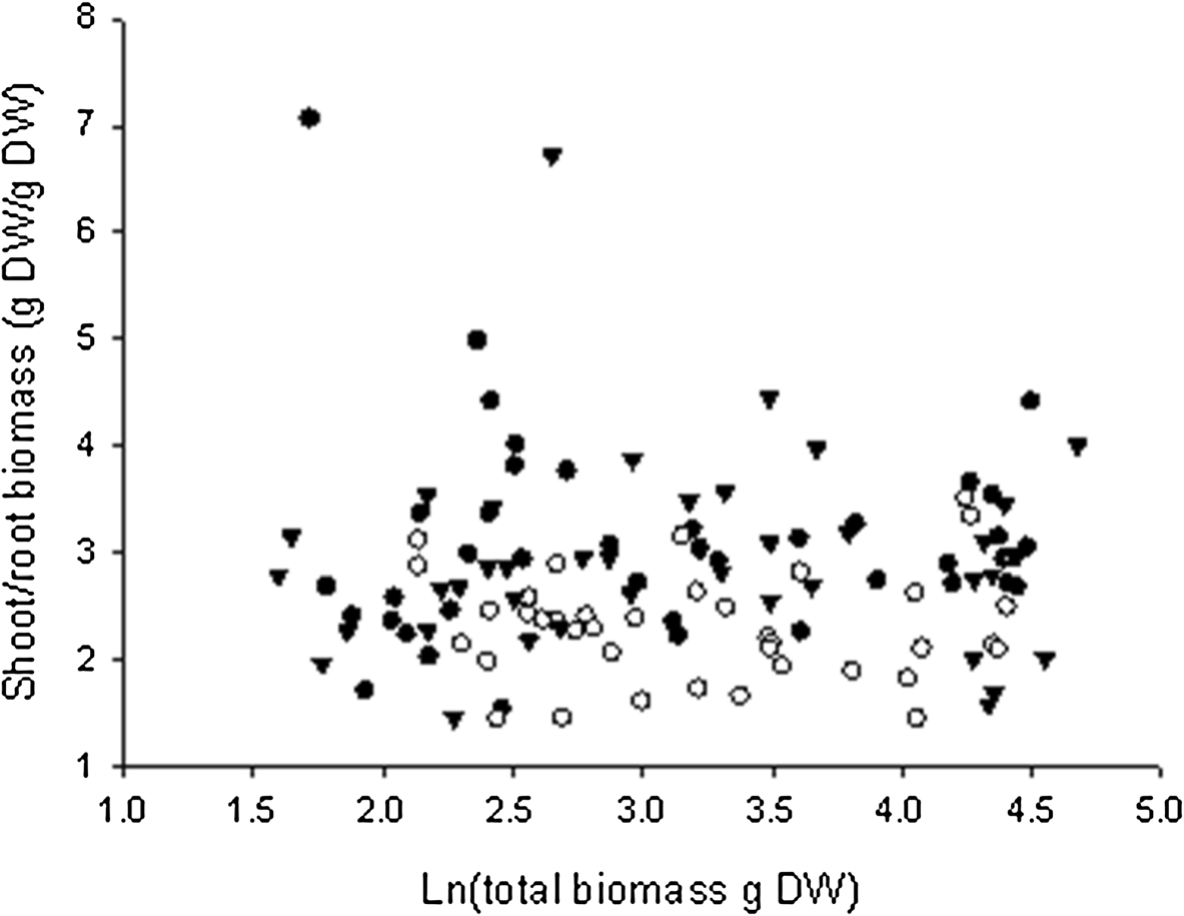
**Hetero-grafting increased shoot to root ratio in comparison to the auto-grafted control.** Biomass allocation between the shoot and the root of Cabernet Sauvignon (CS) hetero-grafted with the rootstocks 1103 Paulsen (closed circles) and Riparia Gloire de Montpellier (closed triangles) and the auto-grafted control (open circles) during the first year of growth.

The rootstocks 1103P and RG increased stem biomass compared to the auto-grafted control at the end of the growth cycle (Figure 1C), i.e. the rootstock genotypes conferred increased vigor to the scion. In addition, the rootstock 1103P increased leaf biomass relative to the auto-grafted control (Figure 1D), whereas the trunk biomass was decreased by the grafting with the rootstock RG (Figure 1B). Root biomass was not significantly different between the three scion/rootstock combinations at the end of the growth cycle (Figure 1A). However, there was a difference in biomass allocation between the shoot and the root (Figure 2). The shoot/root ratio of hetero-grafts was significantly higher than that of the auto-grafted control (Kruskal-Wilk ANOVA on ranks followed by Tukey multiple comparison procedure, data not shown).

**Figure 3:**
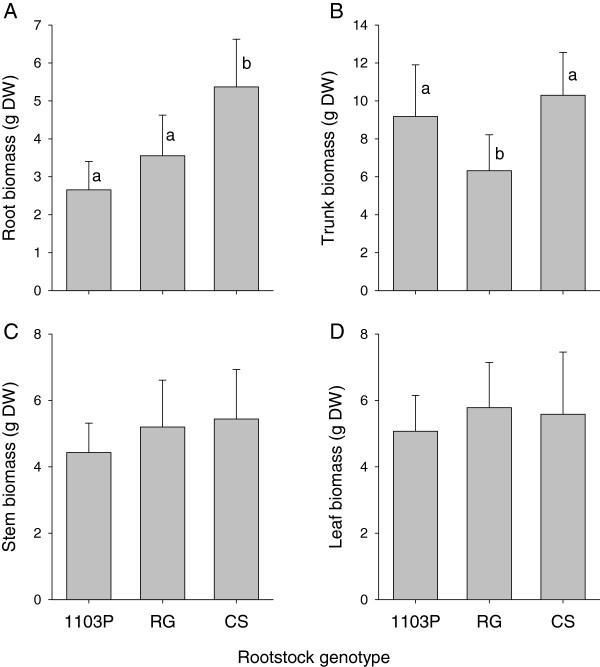
**Rootstock effects on biomass accumulation 4 months after grafting.** Biomass of Cabernet Sauvignon (CS) grafted with the rootstocks 1103 Paulsen (1103P) and Riparia Gloire de Montpellier (RG) and the auto-grafted control (CS) four months after grafting: root **(A)**, trunk **(B)**, stem **(C)** and leaf **(D)** biomass. Means and standard deviations shown, n = 10, results of ANOVA followed by Holm-Sidak multiple comparison procedure indicated by letters where significant differences were observed.

The shoot apex samples were harvested four months after grafting, before differences in scion vigor (stem biomass) became apparent (Figure 3C). Leaf biomass was also unaffected by grafting with rootstocks at the time of apex harvest (Figure 3D). However, the root biomass of the auto-grafted plants was significantly higher than that of the hetero-grafted plants (Figure 3A) and the trunk biomass was significantly lower with the rootstock RG than with the auto-grafted plants and plants grafted onto 1103P (Figure 3B).

### Rootstock effects on gene expression in the shoot apex

**Figure 4:**
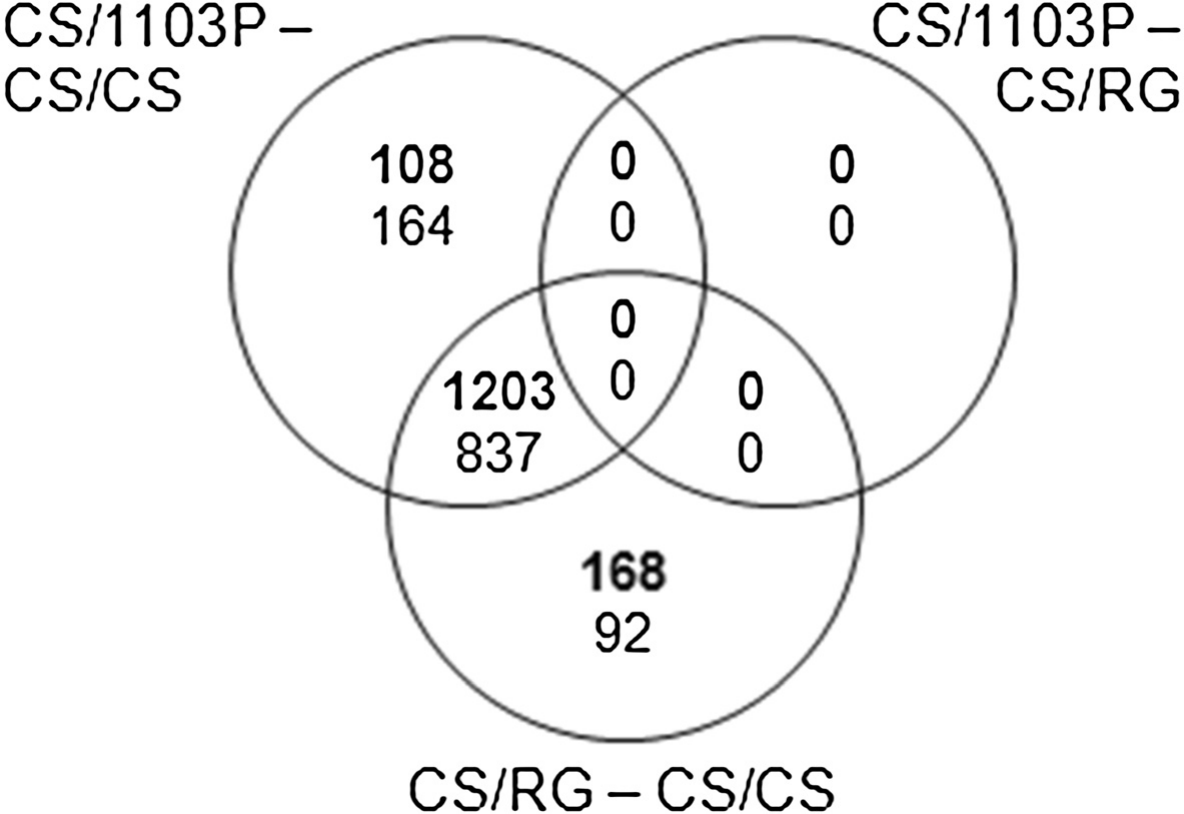
**Venn diagrams showing the genes differentially expressed between auto- and heterografts of grapevine.** Genes differentially expressed in the shoot apex between Cabernet Sauvignon auto-grafted (CS/CS) and grafted with different rootstocks (CS/RG and CS/1103P) are presented in a Venn diagram. Text thickness indicates the direction of gene expression change, the numbers of genes significantly up- and down-regulated are represented by bold and normal text respectively (differential expression defined as a mean log fold change of 1 and p value < 0.05 adjusted with Benjamini-Hochberg).

**Figure 5:**
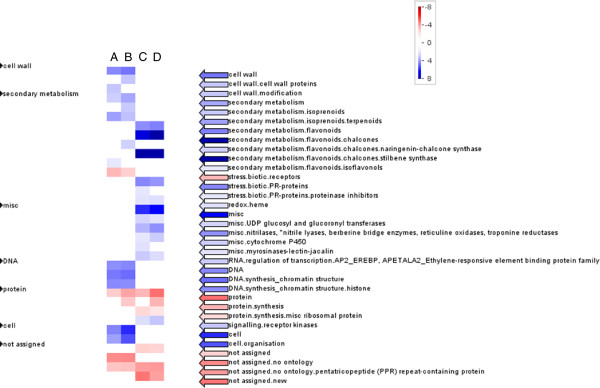
**PageMan display of gene categories enriched in the genes differentially expressed between auto- and heterografts.** Gene category enrichment of genes differentially expressed in the shoot apex of Cabernet Sauvignon after hetero-grafting with the rootstocks Riparia Gloire de Montpellier (RG) and 1103 Paulsen (1103P) in comparison to the auto-grafted control, **A)** genes up-regulated by 1103P, **B)** genes up-regulated by RG, **C)** genes down-regulated by 1103P and **D)** genes down-regulated by RG . Fisher’s exact test (with Bonferroni correction) was used to test whether significantly more genes in a given category were over or under-represented (Color scale is: blue, significant over-representation of genes; red, significant under-representation of genes). In the display, the overrepresented MapMan functional categories are given by collapsing non-significant categories.

Hetero-grafting CS with American rootstock varieties, RG and 1103P, resulted in the differential expression of a large number of genes in the shoot apex when compared to the auto-grafted control CS/CS (Figure 4). In general, the differential expression of most genes was similar in both heterograft combinations: 1203 and 837 genes were up- and down-regulated in both CS/RG and CS/1103P, whereas only 108 and 168 genes were specifically up- and down-regulated in CS/1103P, and only 164 and 92 genes were specifically up- and down-regulated in CS/RG. In fact, no genes were differentially expressed between the two hetero-graft combinations. The normalized expression values, gene expression comparisons, p values, adjusted p values and the gomapman annotation for all significantly differentially expressed genes are given in Additional file 2. The MapMan functional categories (BINs) enriched in the genes differentially expressed between the hetero-grafts and the auto-graft control also highlight the similarity of response of the two hetero-graft combinations (Figure 5, [12-14]).

### Genes up-regulated in the shoot apical meristem by hetero-grafting with rootstocks

The MapMan functional categories [12,13] over-represented in the genes up-regulated in the shoot apex of hetero- compared to auto-grafts belonged to the functional categories cell wall, cell, cell organization, DNA, chromatin structure, histones, LRR receptor kinases, terpenoids and isoflavonols (Table 1 and Figure 5). The GO biological processes DNA replication, DNA metabolic processes and other cellular metabolic processes were also enriched in this gene list, along with the functions helicase, motor, hydrolase and pyrophosphatase activity and the compartments chromosome, organelle, nucleus and extracellular (Table 2).

**Table 1:** MapMan BINs enriched in the genes up-regulated in the shoot apex of hetero-grafts

**Bin number**	**BinName**	**Contingency**	**p value**	**Adusted p-value**
31	Cell	61, 605, 1082, 26089	7.43E-09	2.40E-06
10	Cell wall	47, 509, 1096, 26185	3.75E-06	1.21E-03
31.1	Cell organisation	41, 374, 1102, 26320	2.53E-07	8.16E-05
28	DNA	43, 304, 1100, 26390	1.26E-10	4.09E-08
28.1	DNA synthesis, chromatin structure	34, 190, 1109, 26504	4.81E-11	1.55E-08
28.1.3	DNA synthesis, chromatin structure, histone	15, 35, 1128, 26659	8.46E-10	2.73E-07
16.8.5	Secondary metabolism, flavonoids, isoflavonols	6, 15, 1137, 26679	1.51E-04	4.87E-02
16.1.5	Secondary metabolism, isoprenoids, terpenoids	23, 145, 1120, 26549	4.27E-07	1.38E-04
30.2.3	Signalling, receptor kinases, leucine rich repeat III	8, 28, 1135, 26666	8.55E-05	2.76E-02

MapMan BINs enriched in the genes up-regulated by grafting CS with the rootstocks RG and 1103P in comparison to the auto-grafted control (log fold change of 1, p value <0.05 adjusted with Holm). Contingency gives the number of genes in input list from the BIN, the number of genes on microarray from the BIN, the number of genes from input list not in the BIN and number of genes on microarray not in the BIN.

**Table 2:** GO term enrichment analysis of genes up-regulated in the shoot apex of hetero-grafts

**GO term**	**Ontology**^**a**^	**Description**	**Contingency**	**p-value**	**Adusted p-value**
GO:0006260	P	DNA replication	6, 9, 763, 18735	3.70E-07	1.20E-05
GO:0006259	P	DNA metabolic process	6, 9, 763, 18735	3.70E-07	1.20E-05
GO:0044249	P	Cellular biosynthetic process	6, 16, 763, 18728	2.70E-05	0.00088
GO:0034645	P	Cellular macromolecule biosynthetic process	6, 16, 763, 18728	2.70E-05	0.00088
GO:0009059	P	Macromolecule biosynthetic process	6, 16, 763, 18728	2.70E-05	0.00088
GO:0044260	P	Cellular macromolecule metabolic process	6, 23, 763, 18721	0.00027	0.0086
GO:0044237	P	Cellular metabolic process	207, 3914, 562, 14830	0.00068	0.022
GO:0044238	P	Primary metabolic process	207, 3915, 562, 14829	0.00068	0.022
GO:0003774	F	Motor activity	26, 119, 743, 18625	3.40E-12	1.10E-10
GO:0017111	F	Nucleoside-triphosphatase activity	45, 326, 724, 18418	3.40E-12	1.10E-10
GO:0016818	F	Hydrolase activity, acting on acid anhydrides, in phosphorus-containing anhydrides	45, 326, 724, 18418	3.40E-12	1.10E-10
GO:0016817	F	Hydrolase activity, acting on acid anhydrides	45, 326, 724, 18418	3.40E-12	1.10E-10
GO:0016462	F	Pyrophosphatase activity	45, 326, 724, 18418	3.40E-12	1.10E-10
GO:0004386	F	Helicase activity	19, 207, 750, 18537	0.0011	0.033
GO:0005694	C	Chromosome	67, 277, 702, 18467	3.30E-31	5.90E-30
GO:0043228	C	Non-membrane-bounded organelle	67, 277, 702, 18467	3.30E-31	5.90E-30
GO:0043232	C	Intracellular non-membrane-bounded organelle	67, 277, 702, 18467	3.30E-31	5.90E-30
GO:0043229	C	Intracellular organelle	243, 3945, 526, 14799	2.90E-08	5.20E-07
GO:0043226	C	Organelle	243, 3945, 526, 14799	2.90E-08	5.20E-07
GO:0043227	C	Membrane-bounded organelle	242, 3930, 527, 14814	3.10E-08	5.60E-07
GO:0043231	C	Intracellular membrane-bounded organelle	242, 3930, 527, 14814	3.10E-08	5.60E-07
GO:0005634	C	Nucleus	241, 3922, 528, 14822	3.80E-08	6.90E-07
GO:0005576	C	Extracellular region	86, 1245, 683, 17499	7.00E-06	0.00013
GO:0030312	C	External encapsulating structure	39, 511, 730, 18233	0.00025	0.0044

GO term enrichment of genes significantly up-regulated (log fold change of 1, p value <0.05 adjusted with Holm) in the shoot apex in response to grafting with the rootstocks RG and 1103P in comparison to the auto-grafted control. Contingency gives the number of genes in input list from the GO term, the number of genes on microarray from the GO term, the number of genes from input list not from the GO term and number of genes on microarray not from the GO term.

^a^Key: P, biological process; F, molecular function and C, cellular compartment.

**Figure 6:**
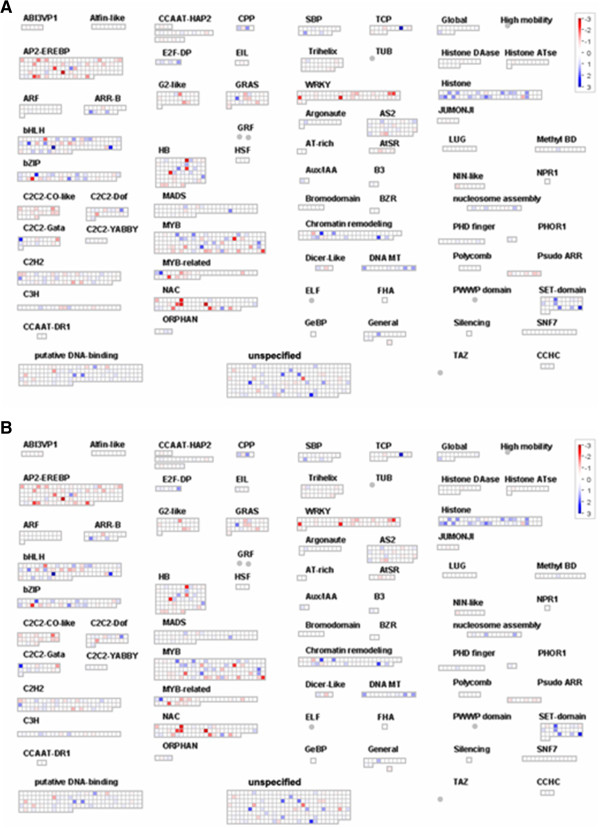
**MapMan transcription overview maps showing differences in transcript levels between auto- and hetero-grafts.** Comparisons made between the shoot apexes of Cabernet Sauvignon auto-grafted and grafted onto the rootstocks 1103 Paulsen **(A)** and Riparia Gloire de Montpellier **(B)**. Up- and down- regulated genes are given in shades of blue and red respectively. The complete set of genes, derived functional categories, normalized expression values and calculated ratios are given in Additional file 2.

Examination of the most strongly up-regulated genes (Additional file 3) highlights the up-regulation of genes associated with DNA and chromatin modification, such as, genes from the category chromatin structure (e.g. *VIT_01s0150g00390*, *VIT_00s0184g00040*, *VIT_07s0005g01430* and *VIT_11s0149g00130*) and genes annotated as being involved in replication control (e.g. *VIT_06s0004g06300*) as well as one SET-domain transcription factor (*VIT_16s0098g01510*) and two chromatin remodeling factors (*VIT_05s0049g00150* and *VIT_04s0023g01610*) (Additional file 3 and Figure 5). In addition to the up-regulation of many genes involved in DNA and chromatin regulation, numerous transcription factors (Figure 6), two sugar transporters (*VIT_14s0030g00240* and *VIT_14s0030g00230*), a developmental gene (*TERMINAL FLOWER-LIKE PROTIEN 1 (TFL1)*, *VIT_06s0080g00290*), and genes involved in amino acid metabolism and cell wall modification were up-regulated (Additional file 3).

### Genes down-regulated in the shoot apical meristem by hetero-grafting with rootstocks

The MapMan functional categories [12,13] over-represented in the genes down-regulated in the hetero- compared to auto-grafts belonged to the functional categories receptor kinases, hormone metabolism (particularly ethylene), secondary metabolism, pathogenesis related (PR) proteins, redox and various miscellaneous genes (Table 3 and Figure 4). The GO biological processes response to stimulus, cell communication and signaling and the functions transferase and oxidoreductase activity was also over-represented (Table 4).

**Table 3:** MapMan BINs enriched in the genes down-regulated in the shoot apex of hetero-grafts

**Bin number**	**BinName**	**Contingency**	**p value**	**Adusted p-value**
17	Hormone metabolism	34, 549, 747, 26507	6.46E-05	1.54E-02
17.5.1	Hormone metabolism, ethylene, synthesis-degradation	10, 78, 771, 26978	1.79E-04	4.26E-02
26	Miscellaneous	106, 1621, 675, 25435	2.49E-14	5.92E-12
26.1	Miscellaneous cytochrome P450	25, 312, 756, 26744	1.15E-05	2.74E-03
26.8	Miscellaneous nitrilases, nitrile lyases, berberine bridge enzymes, reticuline oxidases, troponine reductases	16, 76, 765, 26980	5.48E-09	1.30E-06
26.2	Miscellaneous UDP glucosyl and glucoronyl transferases	22, 263, 759, 26793	2.07E-05	4.92E-03
21.3	Redox heme	5, 6, 776, 27050	6.89E-06	1.64E-03
27.3.3	RNA, regulation of transcription, AP2/EREBP, APETALA2/Ethylene-responsive element binding protein family	14, 104, 767, 26952	5.88E-06	1.40E-03
16.8	Secondary metabolism, flavonoids	25, 239, 756, 26817	1.35E-07	3.22E-05
16.8.2	Secondary metabolism flavonoids, chalcones	17, 40, 764, 27016	1.70E-13	4.06E-11
16.8.2.3	Secondary metabolism flavonoids, chalcones, stilbene synthase	17, 26, 764, 27030	7.42E-16	1.77E-13
30.2	Signalling receptor kinases	38, 637, 743, 26419	7.35E-05	1.75E-02
20.1.7	Stress biotic, PR-proteins	18, 125, 763, 26931	1.15E-07	2.74E-05

MapMan BINs enriched in the genes down-regulated by grafting CS with the rootstocks RG and 1103P in comparison to the auto-grafted control (log fold change of 1, p value <0.05 adjusted with Holm). Contingency as described in Table 1.

**Table 4:** GO term enrichment analysis of genes down-regulated in the shoot apex of hetero-grafts

**GO term**	**Ontology**^**a**^	**Description**	**Contingency**	**p-value**	**Adusted p-value**
GO:0050896	P	Response to stimulus	154, 3959, 375, 14785	0.00032	0.0076
GO:0007154	P	Cell communication	64, 1511, 465, 17233	0.0018	0.044
GO:0023052	P	Signaling	64, 1512, 465, 17232	0.0018	0.044
GO:0016740	F	Transferase activity	147, 3766, 382, 14978	0.00035	0.0088
GO:0016491	F	Oxidoreductase activity	81, 1986, 448, 16758	0.0016	0.039

GO term enrichment of genes significantly down-regulated (log fold change of 1, p value <0.05 adjusted with Holm) in the shoot apex in response to grafting with the rootstocks RG and 1103P in comparison to the auto-grafted control. Contingency as described in Table 2.

^a^Key: P, biological process and F, molecular function.

Examination of the most strongly down-regulated genes (Additional file 4) highlights the importance of the down-regulation of many classes of transcription factors, particularly those belonging to the WRKY class (e.g. *VIT_17s0000g01280*, *VIT_08s0058g01390* and *VIT_04s0008g05760*) (Figure 6). WRKY transcription factors have wide roles in transcriptional activation and repressing plant development and stress responses (as reviewed by [15]).

### Grafting with rootstocks triggers the differential expression of many genes involved in DNA and chromatin modification

Grafting with non-self rootstocks induced the transcriptional reprogramming of many genes associated with chromatin modification. Seventy genes assigned to the functional categories associated with DNA were differentially expressed in the shoot apex in response to grafting with the rootstocks 1103P or RG (Figure 6 and Additional file 5) along with other genes potentially involved in DNA or histone modification (such as SET domain containing proteins and DNA methyltransferases). The histone genes induced by the rootstocks were core histones genes (H2A, H2B, H3 and H4 (Hist1h4h)) except for one centromeric histone (*VIT_15s0046g01110*), whereas a H1 variant, a linker histone, was down-regulated. Two nucleosome assembly proteins were also up-regulated (Additional file 5 and Figure 6); these proteins are chaperones of the core histones H2A and H2B. Five SET domain proteins were also induced by the rootstocks (Additional file 5 and Figure 6); the SET domain is known to have methyltransferase activity targeted to specific lysine residues of histone H3 or H4 [16]. Three DNA methyltransferases were induced by the rootstocks (Additional file 5 and Figure 6); these genes were all belonging to the plant-specific chromomethylase group responsible for asymmetric cytosine methylation in Arabidopsis [17]. Three Werner Syndrome-like exonuclease genes were down-regulated in response to the rootstock genotypes. In Arabidopsis one Werner Syndrome-like exonuclease is known to be involved in post-transcriptional gene silencing [18]. One gene involved in RNA processing (*VIT_12s0059g01120*, a Ribonuclease 3-like/dicer protein) was also down-regulated, this gene is known to be involved in the 24 nt small interfering RNA (siRNA) pathway that modifies chromatin and directs DNA methylation [19].

### Differential expression of hormone related genes

Grafting CS with the rootstocks RG and 1103P triggered the differential regulation of a number of genes associated with hormone metabolism: many genes from the functional categories IAA/auxin and gibberellins were both up- and down-regulated, whereas genes from the category brassinosteroids were generally up-regulated and genes from the categories ABA, salicylic acid and ethylene were generally down-regulated (Additional file 6). Most of the differentially expressed genes from the category auxin were auxin responsive genes, however the expression of three auxin transporters were up-regulated, (three PIN1-like (*VIT_18s0001g15420, VIT_14s0108g00020* and *VIT_08s0040g01230*) and one AUX1-like (*VIT_03s0038g02140*), and one protein ligase involved in auxin signal transduction (*VIT_01s0127g00910*) was down-regulated (Additional file 7). Six genes from the functional category brassinosteroids were up-regulated including two brassinosteroid receptor genes (*VIT_05s0062g01100* and *VIT_16s0100g00710*) (Additional file 8).

### Differential regulation of receptor kinases

Hetero-grafting with rootstocks RG and 1103P resulted in the up-regulation of many LRR containing receptor kinases and the down-regulation of many other classes of receptor kinases such as S-locus glycoprotein like, Domain of Unknown Function (DUF) 26, wall-associated kinase (WAK) and wheat leaf rust resistance LRK10-like receptor kinases (Additional files 8 and 9). Globally, 37 receptor kinases were up- and 63 receptor kinases were down-regulated in the hetero-grafts compared to the auto-graft control (Additional file 8).

## Discussion

### Sampling strategy

Rootstocks are widely used in agriculture and rootstocks can have a profound influence on many aspects of plant development and plant responses to the environment (e.g. as reviewed by [1,2,8]). The sampling strategy selected in this study was designed to reduce the secondary effects of rootstocks on gene expression (such as the indirect effects associated with differences in scion transpiration, water status, photosynthesis, temperature, etc.). Shoot apical meristems were harvested 1 hour before sunrise (harvested under green light) from plants growing outside in well watered pots. The shoot apices were harvested 4 months after grafting when presumably the scions were in a 'steady-state’; the plants had formed successful graft unions, had recovered from the grafting process, had presumably adapted to the presence of a rootstock but had not yet shown significant modifications of scion vigor.

### Rootstock effects on plant development

Rootstocks are known to have a wide range of effects on scion development; the rootstocks used in this work are known to confer differences in scion vigor in the vineyard. The rootstock 1103P increases yields in comparison to the auto-grafted control when grafted with the scion Shiraz, similarly RG increases the yield of the scion Muscat Gordo Blanco in comparison to the auto-grafted control (as reviewed by [3]). In the work presented here on young plants grown in pots, the rootstocks RG and 1103P also affected scion biomass accumulation and root/shoot biomass partitioning during the first growth cycle. Previous work has shown that these rootstocks alter secondary (stem thickness) rather than primary (stem length) shoot growth [20].

In addition to affecting scion development, the rootstocks RG and 1103P affected the partitioning of biomass between the shoot and the root, this relationship was stable throughout plant development. The intrinsic differences in shoot/root biomass allocation are reminiscent of plants responses to a number of environmental factors, such as, nutrient availability, water availability and temperature [21].

### Grafting with rootstocks induced changes in the expression of growth related transcripts

Increased rootstock conferred vigor in apple trees has been associated with an increase in the number of genes up-regulated in the shoot [11]; this was also observed in this work on grapevine in which grafting with vigor increasing rootstocks triggered the up-regulation, rather than the down-regulation, of gene expression. The transcript that best correlated with plant size in grafted apple trees was a sorbitol dehydrogenase [11]; sorbitol is the primary transport form of carbon in apple and Jensen et al. [11] suggested that the shoot apex of vigorous trees were more effective carbon sinks and that this facilitated more vigorous growth. Similarly grafting with rootstocks in grapevine (which increased scion vigor) resulted in the differential regulation of many genes from the functional categories major and minor carbohydrate metabolism and sugar transporters, suggesting that a similar mechanism may exist in grapevine.

The up-regulation of genes from the functional categories cell, cell wall and cell organization could also be associated with the differences in scion growth observed in the hetero-grafted grapevines. The up-regulation of a *TFL 1* gene (*VIT_06s0080g00290*) could be related to the non-self rootstock mediated increase in scion vegetative growth. In apple trees, the silencing of *MdTFL1* is associated with decreased vegetative growth and reduced generation time [22]. The grapevine homologue of TFL1 is also known to delay flowering when over-expressed in Arabidopsis and tobacco [23].

### Grafting with rootstocks triggers the differential expression of many genes involved in DNA and chromatin modification

In eukaryotes epigenetic mechanisms can regulation chromatin structure and gene expression, for example by DNA methylation, histone modification and certain aspects of the siRNA signaling pathway [24]. DNA methyltransferases add a methyl group to a cytosine base to produce 5 methylcytosine; DNA methylation is thought to repress transposons activity and in some cases gene expression [25]. The up-regulation of genes associated with DNA methylation could be associated with the repression of transposon activity or gene expression in hetero-grafted plants. The up-regulation of many histones could also be related to alterations in the chromatin structure of hetero-grafted plants and could be involved in some of the gene expression differences observed between the hetero- and auto-grafted plants. Both DNA methylation and histone modification can be affected by siRNAs [24] and small RNAs are known to be graft transmissible in plants (e.g. [26]). We propose the hypothesis that grafting with non-self rootstocks alters the small RNA population of the scion and mediates epigenetics changes in the recipient tissue. However, chromatin modification is known to be induced in response to various stresses such as salt, drought and cold stress as well as being involved in a number of hormone signaling cascades (as reviewed by [27]). Therefore, the differential expression of genes in response to hetero-grafting could be an indirect response of chromatin modification genes via grafting-related stress responses and/or a direct consequence of siRNA trafficking between the rootstock and scion. In addition to possible epigenetic regulation induced by hetero-grafting, both post-transcriptional and post-translational modifications are also possible. Numerous genes involved in protein synthesis, degradation and targeting were differentially expressed in the hetero-grafts. Furthermore, six genes involved in RNA processing and three genes involved in RNA binding were up-regulated in the hetero-grafts (the only down-regulated gene was the ribonuclease 3-like/dicer protein previously mentioned).

### The differential expression of receptor kinases in response to hetero-grafting with a non-self rootstock

Plant genomes contain large numbers of receptor kinases with very divergent extracellular domains. The receptor kinases that were differentially expressed in response to grafting with non-self rootstocks included genes from the functional categories LRR, S-locus glycoprotein like, DUF 26, WAK and LRK10-like. LRR proteins are the largest group of receptor kinases in plants and the motif is thought to be involved in signal transduction and to mediate protein-protein interactions. In this study, generally the receptor kinases from the LRR family were up-regulated in the shoot apex of the hetero-grafts (14 genes were up and only 4 genes were down-regulated). LRR domain containing proteins have been implicated in many developmental pathways and defense responses (as reviewed by [28]); this could imply that grafting with rootstocks triggered a defense response throughout the plant. Similarly, LRK10 receptor kinases were down-regulated in the hetero-grafts and LRK10 was first identified as a leaf rust resistance gene in wheat.

S-locus glycoprotein-like receptor kinases were first identified as being important in self-incompatibility responses in Brassica flowers and have since been shown to be involved in plant defense responses (as reviewed by [29]). Surprisingly, the S-locus receptor kinases were also down-regulated, rather than up-regulated as we would have expected. However, at least one S-locus receptor kinase in Arabidopsis is a negative regulator of plant defense responses [30] suggesting that the interpretation of differential expression of receptor kinases is more complicated that it may first appear. However, it is possible that the S-locus receptor kinases differentially expressed in response to grafting with a non-self rootstock genotype in order to suppress a self-incompatibility response throughout the plant.

### The differential expression of hormone signaling related genes in response to hetero-grafting with a non-self rootstock

The differential regulation of genes from the functional category hormones could suggest the involvement of hormone signaling between the two different genotypes in a grafted plant. In agreement with this observation, differences in hormone signaling and the sequestration of hormones in the rootstock shank (particularly auxin, gibberellins, ABA and cytokinins) have been proposed as mechanisms of rootstock control of scion growth (as reviewed by [2]).

Many genes from the functional category brassinosteroids were up-regulated; brassinosteroids are known to interact with plant defense receptor signaling pathways and modulate signaling of the tradeoff between growth and plant immune responses [31]. This could suggest the grafting with a non-self rootstock alters defense responses in the scion. This idea is further supported by the down-regulation of genes from the functional categories ABA, ethylene and salicylic acid, which could also be associated with plant defense response pathways [32].

### Overlap between genes differentially expressed in the shoot apex and genes associated with hybrid vigor

Hybrid vigor is the phenomenon in which hybrids have superior performances over their parents in a number of traits such as biomass accumulation, growth rate and fertility (e.g. [33]). The expression of circadian oscillator genes has been associated with hybrid vigor in Arabidopsis [34,35], although clock genes were not differentially expressed in this experiment, this could be related to the time of day of the harvest and the culture of plants outside under natural conditions. Shen et al. [35] showed that DNA methylation is increased in hybrids across the whole genome, but especially in the transposable elements. Seventy-seven genes sensitive to methylome remodeling were identified as being differentially repressed in the hybrids, including the down-regulation of genes involved in flavonoid biosynthesis and the up-regulation of two genes involved in auxin signaling (as well as the up-regulation of auxin transport). In the shoot apex, grafting with vigor increasing rootstocks also resulted in the up-regulation of many genes involved in DNA and chromatin modification and the down-regulation of many genes involved in flavonoid biosynthesis. In addition, many genes involved in auxin transport, signal transduction and auxin responsive genes were differentially expressed in response to grafting with rootstocks. This could suggest that grafting with vigor increasing rootstocks induces a transcriptional response similar to that of hybrid vigor.

## Conclusions

Grafting *V. vinifera* with rootstocks originating from American *Vitis spp*. had a profound effect on scion gene expression in the shoot apex. However, the choice of rootstock genotype did not appear to have a dramatic effect on gene expression; this could suggest that auto- and hetero-grafting was the major factor in the regulation gene expression in the shoot apex. The functional categories over-represented in the rootstock responding gene lists (such as chromatin regulation, cell organization and hormone signaling) could suggest that there is some degree of self- and non-self root recognition. Many genes differentially expressed in the shoot apex between hetero- and auto-grafted plants are also known to be involved in defense responses supporting the idea that the scion can detect the presence of a non-self rootstock. The similarity between the transcriptional response at the shoot apex to vigor increasing non-self rootstocks and in the shoot of vigorous hybrids could suggest that similar mechanisms are involved.

## Methods

### Plant material

Plant material, grafting procedure and growing conditions were as described by [20]. Briefly, CS, *V. riparia* cv. 'Riparia Gloire de Montpellier’ (RG) and the *V. berlandieri* x *V. rupestris* hybrid cv. '1103 Paulsen’ (1103P) hardwood was grafted in March using mechanical omega grafting. After callusing for 28 days at 28°C and 90% humidity, the plants were transferred to a greenhouse for one month and then transferred to 7 L pots filled with calcareous clay soil and cultivated in an experimental plot outside watered with nutrient solution.

### Calculation of thermal time

In most studies of plant growth conducted in non- or semi-controlled environmental conditions, time is expressed as thermal time (degree days), in which temperature dependent variations in growth rate are compensated for. Thermal time is based upon the linear relationships between the rates/durations of growth within a non-deleterious range of air temperatures and is calculated by incrementing mean daily air temperature minus a threshold temperature. In this study, we have used the standard threshold temperature for grapevine shoot growth [36]. Thermal time was calculated by the daily integration of mean air temperature minus a base temperature of 10°C. It was expressed in degree days (°Cd) and was calculated from the moment the grafted plants were planted into pots and transferred to the experimental plot. The climatic conditions from 1st June until 30th September 2010 were: average air temperature 20.6°C; the average daily mean global radiation was 1962 joules cm^-2^; the average relative humidity 68.1%.

### Biomass measurements

Ten plants per scion/rootstock combination were separated into leaf, stem, root and trunk samples on four dates during the first growth cycle after grafting (06/07/2010, 26/07/2010, 17/08/2010 and 15/09/2010 corresponding to 466, 724, 932 and 1246 °Cd respectively). Samples were dried in an oven at 80°C until the samples reached a constant weight.

### Statistical analysis of biomass data

All biomass data were analyzed using Sigma Plot 11.

### RNA extraction

Four pools of 5 shoot apex zones (approximately 4 mm in length) were harvested from 20 single-stemmed grapevines (i.e. one apex per plant) at the end of the night for each scion/rootstock combination in July (4 months after grafting) and immediately snap frozen in liquid nitrogen. Total RNA was extracted using the Spectrum Plant Total RNA kit (Sigma-Aldrich) according to the manufacturer’s instructions.

### Microarray analysis

The microarrays used were the grape whole genome microarrays from Nimblegen, Roche, (Design name 090918 Vitus exp HX12). The microarray probe design is based on the 12X genome assembly [37] using the grapevine V1 gene model prediction from CRIBI [38]. The probe design is available online [39]. The microarray contains 118015 probes with an average of 4 probes per gene; the expression of 29549 genes can be quantified with the microrarray. The correspondence between probe identifiers and gene identifiers were obtained from [38]. The microarray hybridisations were done for the 12 samples (three scion/rootstock combinations with four biological replicates) by the Plateforme Biopuces, Institut National des Sciences Appliquées, Toulouse, France according to the manufacturer’s instructions.

The microarray data were analyzed using the statistical package R, version 2.14.0 [40] with various Bioconductor packages [41,42]). Microarray quality controls were performed using the arrayQualityMetrics package [43]. Expression intensities were background corrected, quantile-normalized and summarized using the rma function of the oligo package [44]. The dataset supporting the results of this article is available from ArrayExpress [45]; the accession number is E-MTAB-1523 [46]. Differentially expressed genes were identified using the limma package [47]; genes with absolute log2 fold changes >1 and Benjamini-Hochberg corrected p-values below 0.05 were considered significant.

Differences in gene expression were visualized using MapMan [12,13] and PageMan [14]. The MapMan mapping file was obtained from [48]; 27837 of the 29549 genes on the microarray are present in the mapping file. Enrichments of functional categories of the MapMan annotation in the significantly differentially expressed genes were tested for significance by applying Fisher tests with a Bonferroni correction for multiple tests using Mefisto Version 0.23beta [49], the contingency data are given as follows: genes from functional category in input list, genes from functional category on microarray, background input list and background microarray. Enrichment of Gene Ontology (GO) terms in significantly differentially expressed genes was evaluated using analysis tool from AgriGO [50,51] with Fisher tests and Bonferroni multiple testing correction (p < 0.05).

### qPCR analysis

For qPCR experiments, genomic DNA contamination was removed from the RNA with the Turbo DNA-free kit from Ambion (according to the manufacturer’s instructions) and reverse transcription was done using the Superscript III kit from Invitrogen (using oligo dT primers, 1.5 μg RNA and according to the manufacturer’s instructions). Gene expression was analyzed on a Biorad CFX96 machine using iQ Sybr Green Supermix (according to the manufacturer’s instructions). The quality (and quantity) of cDNA synthesized was tested using two sets of primers that amplified the 3’ and 5’ regions of the same reference gene (a SAND protein, *VIT_06s0004g02820*) and genomic DNA contamination was checked by qPCR using intron specific genes (Additional file 10). The expression of genes of interest was normalized with *VIT_06s0004g02820* and two additional reference genes were used to confirm the stability of expression of *VIT_06s0004g02820* (Additional file 10). The relative expression is presented as 40 minus delta Ct using *VIT_06s0004g02820* as the reference gene. PCR efficiency for each primer pair was calculated using LinRegPCR [52]. Nine genes were selected for qPCR analysis based upon their degree of up- or down-regulation and the potential interest of their putative function.

## Competing interests

The authors declare that they have no competing interests.

## Authors’ contributions

SJC carried out the data acquisition, analysis and interpretation, and drafted the manuscript. NO made substantial contributions to the conception and design of the experiments as well as the manuscript preparation. Both authors approved and read the final manuscript.

## Supplementary Material

Additional file 1 **Validation of microarray data (open bars) by qPCR (filled bars) in the shoot apical meristem of Cabernet Sauvignon auto-grafted (CS) and grafted with the rootstocks Riparia Gloire de Montpellier (RG) and 1103 Paulsen (1103P): *VIT_11s0149g00130* (A), *VIT_07s0191g00240* (B), *VIT_15s0048g02430* (C), *VIT_18s0001g10150* (D), *VIT_19s0014g03130* (E), *VIT_06s0080g00290* (F), *VIT_16s0098g01510* (G), *VIT_09s0002g03160* (H) and *VIT_18s0001g03180* (I).** Means and standard deviations shown, n = 3. Click here for file

Additional file 2 All genes differentially expressed (log fold change of 1, p value <0.05 adjusted with Benjamini-Hochberg) in the shoot apex of Cabernet Sauvignon (CS) hetero- (CS/Riparia Gloire de Montpellier (RG) or CS/1103 Paulsen (1103P)) compared to auto-grafts (CS/CS). Click here for file

Additional file 3 Genes strongly up-regulated (log fold change of >2, p value <0.05 adjusted with Benjamini-Hochberg) in the shoot apex of Cabernet Sauvignon (CS) hetero- (CS/Riparia Gloire de Montpellier (RG) and CS/1103 Paulsen (1103P)) compared to auto-grafts (CS/CS). Click here for file

Additional file 4 Genes strongly down-regulated (log fold change of >2, p value <0.05 adjusted with Benjamini-Hochberg) in the shoot apex of Cabernet Sauvignon (CS) hetero- (CS/Riparia Gloire de Montpellier (RG) and CS/1103 Paulsen (1103P)) compared to auto-grafts (CS/CS). Click here for file

Additional file 5 Genes associated with DNA differentially expressed (log fold change of 1, p value <0.05 adjusted with Benjamini-Hochberg) in the shoot apex of Cabernet Sauvignon (CS) hetero- (CS/Riparia Gloire de Montpellier (RG) or CS/1103 Paulsen (1103P)) compared to auto-grafts (CS/CS). Click here for file

Additional file 6 **MapMan hormone regulation overview maps showing differences in transcript levels between the shoot apexes of Cabernet Sauvignon auto-grafted and grafted onto the rootstocks 1103 Paulsen (A) and Riparia Gloire de Montpellier (B).** Up- and down-regulated genes are given in shades of blue and red respectively. The complete set of genes, derived functional categories, normalized expression values and calculated ratios are given in Additional file 7. Click here for file

Additional file 7 Genes associated with hormone signaling differentially expressed (log fold change of 1, p value <0.05 adjusted with Benjamini-Hochberg) in the shoot apex of Cabernet Sauvignon (CS) hetero- (CS/Riparia Gloire de Montpellier (RG) or CS/1103 Paulsen (1103P)) compared to auto-grafts (CS/CS). Click here for file

Additional file 8 Genes associated with receptor kinases differentially expressed (log fold change of 1, p value <0.05 adjusted with Benjamini-Hochberg) in the shoot apex of Cabernet Sauvignon (CS) hetero- (CS/Riparia Gloire de Montpellier (RG) or CS/1103 Paulsen (1103P)) compared to auto-grafts (CS/CS). Click here for file

Additional file 9 **MapMan receptor kinase overview maps showing differences in transcript levels between the shoot apexes of Cabernet Sauvignon auto-grafted and grafted onto the rootstocks 1103 Paulsen (A) and Riparia Gloire de Montpellier (B).** Up- and down-regulated genes are given in shades of blue and red respectively. The complete set of genes, derived functional categories, normalized expression values and calculated ratios are given in Additional file 8. Click here for file

Additional file 10 Sequence and mean PCR efficiency of primers used for qPCR analysis. Click here for file
