# Supplementary material for: Grafting with rootstocks induces extensive transcriptional re-programming in the shoot apical meristem of grapevine
Source: BMC Plant Biol. 2013 Oct 2;13:147. doi: 10.1186/1471-2229-13-147 (PMC3852942; doi:10.1186/1471-2229-13-147)
Supplement: Additional file 10 — Sequence and mean PCR efficiency of primers used for qPCR analysis. [file 1471-2229-13-147-S10.docx]

Additional File 10. Sequence and mean PCR efficiency of primers used for qPCR analysis.

| **Gene code** | **Gene function** | **Use of primer** | **Orientation of primer** | **Primer sequences ('5-'3)** | **Mean PCR efficiency** |
| --- | --- | --- | --- | --- | --- |
| VIT_06s0004g02820 | SAND protein | 5' test | Forward | ACCCCTTTGCTCGGAGGAACAGAT | 2.028 |
|  |  |  | Reverse | ACCTGAAGCTTGCCTTGTCGCA |  |
| VIT_06s0004g02820 | SAND protein | 3' test | Forward | TGCTGGGTTACCCCGGAGTTTGA | 1.967 |
|  |  |  | Reverse | CAGACCCGGTTGCACGTCCG |  |
| VIT_06s0004g02820 | SAND protein | genomic DNA test | Forward | CTGTGCACCTGCTTCGCCCA | 1.954 |
|  |  |  | Reverse | AGGAATGGGGAGAGGGGTCCT |  |
| VIT_02s0025g01050 | Ubiquitin conjugating enzyme E2-17 | Reference gene | Forward | TCCTCCTGACAGTCCATATGCTGGT | 1.87 |
|  |  |  | Reverse | GGGCTGGGCTCCACTGCTCC |  |
| VIT_14s0171g00440 | Glyceraldehyde-3-phosphate dehydrogenase | Reference gene | Forward | GCTGGAATTGCTCTGAATGA | 1.912 |
|  |  |  | Reverse | GCTGAAAATTCTGGGTCCAA |  |
| VIT_19s0014g03130 | Auxin.induced-regulated-responsive-activated protein | Gene of interest | Forward | TCCAGGATCAACTCCATGCCTCGT | 1.978 |
|  |  |  | Reverse | GCAGGAAAGAGCAGGCGGGT |  |
| VIT_15s0048g02430 | Gene involved in ethylene signaling | Gene of interest | Forward | GCCCGGTTTGCAGGTGCTCA | 1.98 |
|  |  |  | Reverse | ACGGCCCTATGGACCGCACT |  |
| VIT_11s0149g00130 | Gene involved in DNA synthesis and chromatin structure | Gene of interest | Forward | TGGATGCAGCGAGGTCTGGA | 2.048 |
|  |  |  | Reverse | GCCGTCTCTCCGATATGTGGCT |  |
| VIT_07s0191g00240 | Basic Helix-Loop-Helix family transcription factor | Gene of interest | Forward | CTTGGCTCCACTCCGTCCGC | 1.947 |
|  |  |  | Reverse | TCCTTGGATCCTCCCCACCGT |  |

| **Gene code** | **Gene function** | **Use of primer** | **Orientation of primer** | **Primer sequences ('5-'3)** | **Mean PCR efficiency** |
| --- | --- | --- | --- | --- | --- |
| VIT_18s0001g10150 | APETALA2_Ethylene-responsive element binding protein transcription factor | Gene of interest | Forward | GGCCGCTCTGCTCGCCTCAA | 2.069 |
|  |  |  | Reverse | GGGCGGTCTCCATGGCGTCAA |  |
| VIT_06s0080g00290 | Terminal flower-like protein 1 | Gene of interest | Forward | GGCCCCAGCGACCCATACCT | 1.92 |
|  |  |  | Reverse | TGGGTTGACTGTTTGTCGGCGT |  |
| VIT_09s0002g03160 | Poly(A)-binding protein | Gene of interest | Forward | TCCCTCACCCAAATCCTACCCCA | 1.927 |
|  |  |  | Reverse | TGGGGCAAAAGGGCGCATCA |  |
| VIT_16s0098g01510 | SET-domain transcriptional regulator family | Gene of interest | Forward | AGAAGGGTTCACAGTTGAGGCAGA | 1.931 |
|  |  |  | Reverse | ACCACGCTTGTCAGGACAGATGA |  |
| VIT_18s0001g03180 | Nodulin-like protein | Gene of interest | Forward | AGGCCCAGTGTTTGTGACAGCA | 1.951 |
|  |  |  | Reverse | CCCATGCCACAGAGTAAAGGCCG |  |
