# Supplementary material for: Grafting with rootstocks induces extensive transcriptional re-programming in the shoot apical meristem of grapevine
Source: BMC Plant Biol. 2013 Oct 2;13:147. doi: 10.1186/1471-2229-13-147 (PMC3852942; doi:10.1186/1471-2229-13-147)
Supplement: Supplementary file 8 [file 1471-2229-13-147-S1.docx]

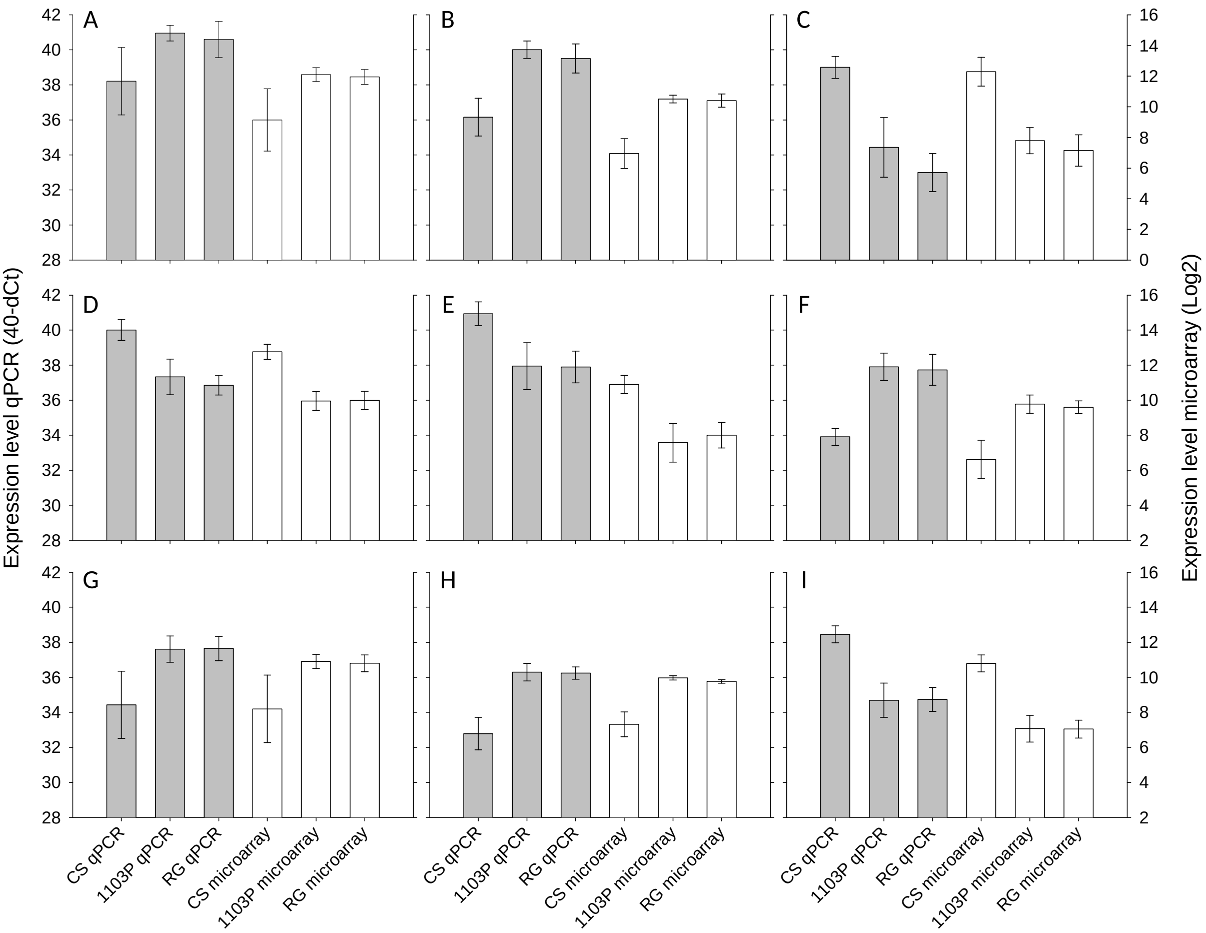

A
B
C
D
E
F
G
H
I
Expression level qPCR (40-dCt)
Expression level microarray (Log2)
42
40
38
36
34
32
30
28
16
14
12
10
8
6
4
2
0
CS qPCR
1103P qPCR
RG qPCR
CS microarray
1103P microarray
RG microarray
