# Supplementary material for: Grafting with rootstocks induces extensive transcriptional re-programming in the shoot apical meristem of grapevine
Source: BMC Plant Biol. 2013 Oct 2;13:147. doi: 10.1186/1471-2229-13-147 (PMC3852942; doi:10.1186/1471-2229-13-147)
Supplement: Supplementary file 9 [file 1471-2229-13-147-S6.docx]

A

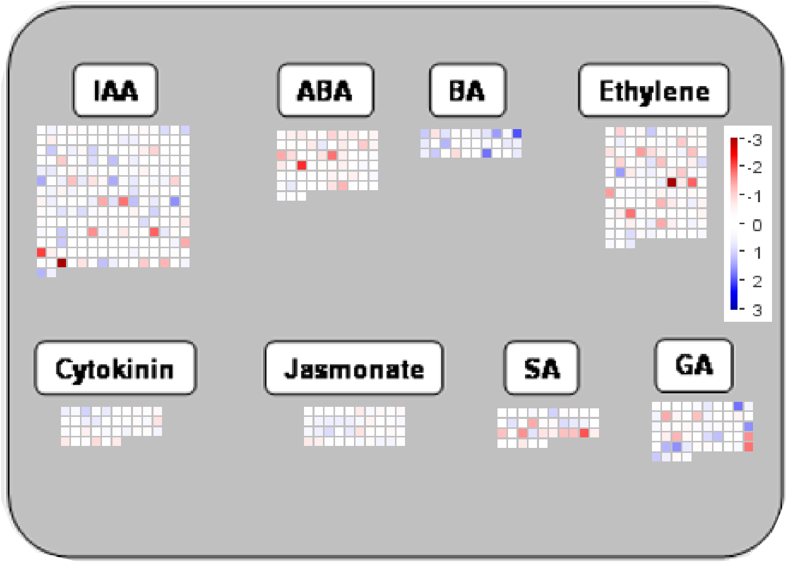
IAA
ABA
BA
Ethylene
-3
-2
-1
0
1
2
3
Cytokinin
Jasmonate
SA
GA

B

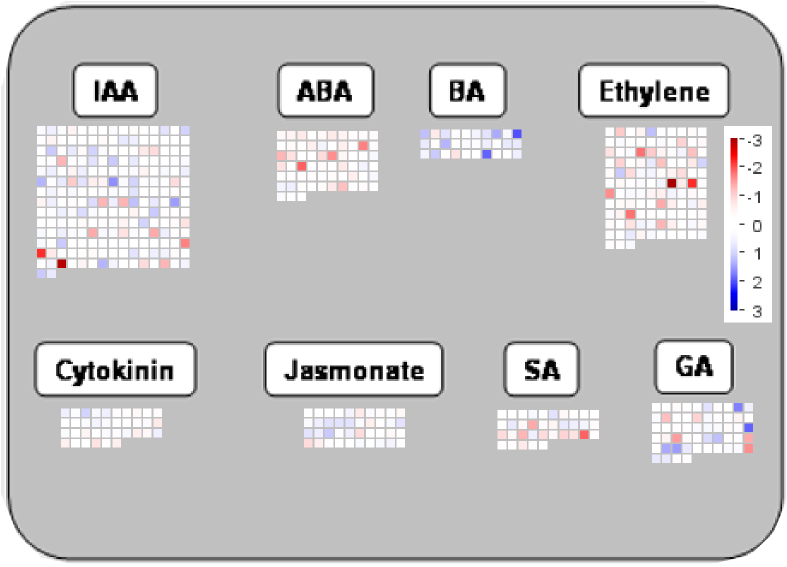
IAA
ABA
BA
Ethylene
-3
-2
-1
0
1
2
3
Cytokinin
Jasmonate
SA
GA
