# Supplementary material for: Grafting with rootstocks induces extensive transcriptional re-programming in the shoot apical meristem of grapevine
Source: BMC Plant Biol. 2013 Oct 2;13:147. doi: 10.1186/1471-2229-13-147 (PMC3852942; doi:10.1186/1471-2229-13-147)
Supplement: Supplementary file 10 [file 1471-2229-13-147-S9.docx]

A

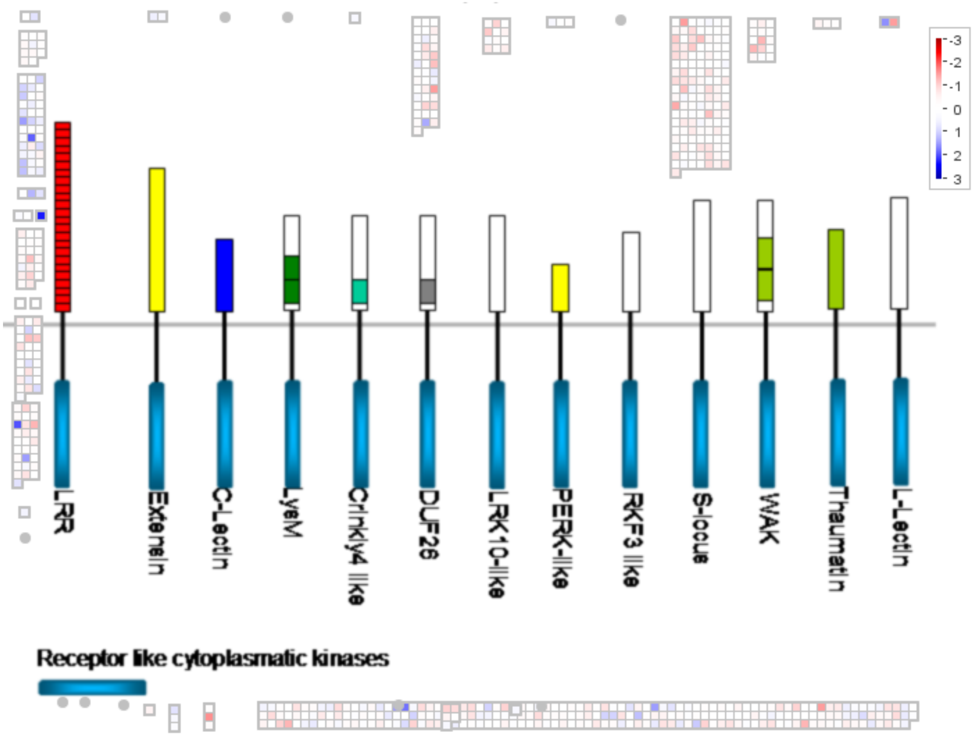
-3
-2
-1
0
1
2
3
LRR
Extensin
C-Lectin
LysM
Crinkly4 like
DUF26
LRK10-like
PERK-like
RKF3 like
S-locus
WAK
Thaumatin
L-Lectin
Receptor like cytoplasmatic kinases

B

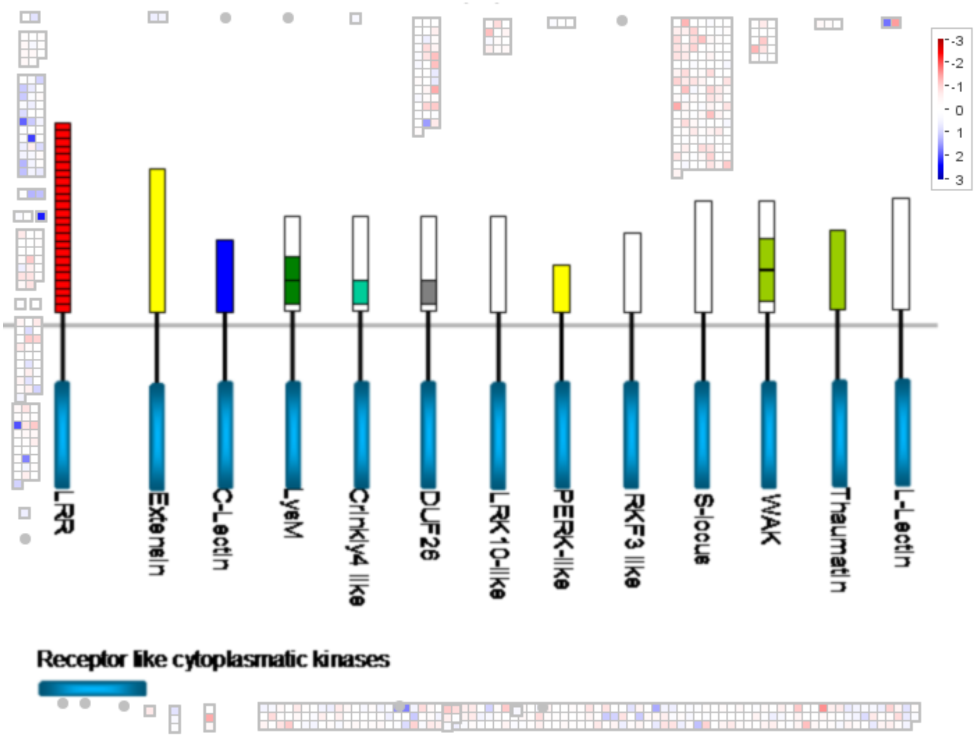
-3
-2
-1
0
1
2
3
LRR
Extensin
C-Lectin
LysM
Crinkly4 like
DUF26
LRK10-like
PERK-like
RKF3 like
S-locus
WAK
Thaumatin
L-Lectin
Receptor like cytoplasmatic kinases
